# (*Z*)-Ethyl 2-(2,4-dimethyl­benzyl­idene)-7-methyl-3-oxo-5-phenyl-3,5-dihydro-2*H*-thia­zolo[3,2-*a*]pyrimidine-6-carboxyl­ate

**DOI:** 10.1107/S1600536811052925

**Published:** 2011-12-14

**Authors:** Xiao-Yan Chen, Han-Chu Wang, Qian Zhang, Zhi-Jian Song, Fei-Yun Zheng

**Affiliations:** a The First Affiliated Hospital, Wenzhou Medical College, Wenzhou, Zhejiang Province 325035, People’s Republic of China; b Institute of Biomedical Informatics/Zhejiang Provincial Key Laboratory of Medical Genetics, Wenzhou Medical College, Wenzhou, Zhejiang Province 325035, People’s Republic of China

## Abstract

In the title compound, C_25_H_24_N_2_O_3_S, the dihedral angles between the thia­zole ring and the phenyl and substituted benzene rings are 84.91 (11) and 11.58 (10)°, respectively. The dihydro­pyrimidine ring adopts a flattened boat conformation. The olefinic double bond is in a *Z* configuration.

## Related literature

For related structures, see: Kulakov *et al.* (2009 ▶); Zhao *et al.* (2011 ▶). For background to the biological properties of fused pyrimidine derivatives, see: Al-Rashood & Abdel-Aziz (2010 ▶); Ashok *et al.* (2007 ▶); Jang *et al.* (2011 ▶); Wichmann *et al.* (1999 ▶).
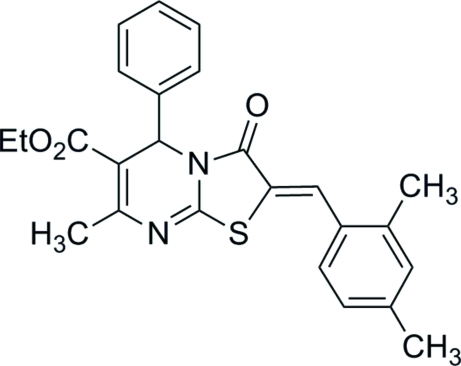


## Experimental

### 

#### Crystal data

C_25_H_24_N_2_O_3_S
*M* *_r_* = 432.52
Monoclinic, 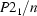

*a* = 9.690 (5) Å
*b* = 10.620 (5) Å
*c* = 21.692 (12) Å
β = 90.682 (10)°
*V* = 2232 (2) Å^3^
*Z* = 4
Mo *K*α radiation
μ = 0.17 mm^−1^
*T* = 293 K
0.32 × 0.27 × 0.16 mm

#### Data collection

Bruker SMART CCD area-detector diffractometer
Absorption correction: multi-scan (*SADABS*; Bruker, 2002 ▶) *T*_min_ = 0.675, *T*_max_ = 1.000
8761 measured reflections
4257 independent reflections
3096 reflections with *I* > 2σ(*I*)
*R*_int_ = 0.026

#### Refinement

*R*[*F* ^2^ > 2σ(*F* ^2^)] = 0.050
*wR*(*F* ^2^) = 0.155
*S* = 1.01
4257 reflections
284 parameters
H-atom parameters constrained
Δρ_max_ = 0.31 e Å^−3^
Δρ_min_ = −0.26 e Å^−3^

### 

Data collection: *SMART* (Bruker, 2002 ▶); cell refinement: *SAINT* (Bruker, 2002 ▶); data reduction: *SAINT*; program(s) used to solve structure: *SHELXS97* (Sheldrick, 2008 ▶); program(s) used to refine structure: *SHELXL97* (Sheldrick, 2008 ▶); molecular graphics: *SHELXTL* (Sheldrick, 2008 ▶); software used to prepare material for publication: *SHELXTL*.

## Supplementary Material

Crystal structure: contains datablock(s) I, global. DOI: 10.1107/S1600536811052925/rn2098sup1.cif

Structure factors: contains datablock(s) I. DOI: 10.1107/S1600536811052925/rn2098Isup2.hkl

Supplementary material file. DOI: 10.1107/S1600536811052925/rn2098Isup3.cml

Additional supplementary materials:  crystallographic information; 3D view; checkCIF report

## Figures and Tables

**Fig. 1.**
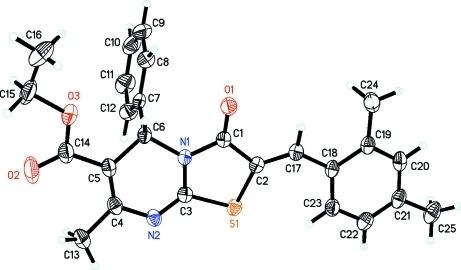
The molecular structure of the title compound, showing 30% displacement ellipsoids for the non-hydrogen atoms. Hydrogen atoms are drawn as spheres of arbitrary radius.

## supplementary crystallographic information

### Comment

Pyrimidine has gained considerable attention because of its diversity in biological activity, such as anticarcinogenic and analgesic properties (Ashok *et al.*, 2007). Thiazoles and their derivatives are also found to be associated with various biological activities such as antibacterial, antifungal and anti-inflammatory properties (Jang *et al.*, 2011). Furthermore, the pyrimidines and thiazoles rings are found in the skeleton of many compounds with potent biological activity (Al-Rashood *et al.*,2010; Wichmann, *et al.*, 1999). Since the two heterocyclic moieties constitute two active pharmacophores that are highly active against inflammation and pain, combining the two is expected to have a synergistic effect in dealing with diseases.

In this paper, we report the molecular and crystal structure of (*Z*)-ethyl 2-(2,4-dimethylbenzylidene)-7-methyl-3-oxo-5-phenyl-3,5-dihydro-2*H*-thiazolo [3,2-*a*]pyrimidine-6-carboxylate (I). The molecule (I), consists of one thiazole ring and two benzene rings. The fused pyrimidine ring has usual geometry as observed in other fused pyrimidine compounds (Kulakov, *et al.*, 2009; Zhao, *et al.*, 2011). The thiazole ring makes dihedral angles of 84.91 (11) and 11.58 (10)° with the benzene rings C7—C12 and C18–C23, respectively. The pyrimidine ring adopts a flattened boat conformation. The C2—C17 double bond exists in the *Z* configuration. The molecular structure of (I) is illustrated in Fig. 1.

### Experimental

In a one pot Biginelli reaction, a mixture of 5 mmol of benzaldehyde, 6 mmol ethyl acetoacetate, 7.5 mmol thiourea and 10 ml of EtOH was stirred at 50°C in presence of sulfamic acid catalyst for 3 h to obtain 6-methyl-4-phenyl-2-thioxo-1,2,3,4- tetrahydropyrimidine-5-carboxylate. Then the product (2 mmol) was reacted with ethyl chloroacetate (2 mmol) in the presence of pyridine for 4 h; and 2,4-dimethylbenzaldehyde (2 mmol) and piperidine were added, and the mixture refluxed for 4 h until the TLC assay indicated that the reaction was completed. The reaction mixture was cooled and filtered to give crude product. The solid was collected and crystallized from acetic acid to obtain the final product. Single crystals of the title compound were grown in a CH_2_Cl_2/_CH_3_OH mixture (2:1 *v*/*v*) by slow evaporation.

### Refinement

The H atoms were positioned geometrically (C—H = 0.93 and 0.96 Å) and refined as riding with *U*iso(H) = 1.2*U*eq(C) or 1.5*U*eq(methyl C).

### Crystal data

**Table:**

C_25_H_24_N_2_O_3_S	*F*(000) = 912
*M**_r_* = 432.52	*D*_x_ = 1.287 Mg m^−^^3^
Monoclinic, *P*2_1_/*n*	Mo *K*α radiation, λ = 0.71073 Å
*a* = 9.690 (5) Å	Cell parameters from 2729 reflections
*b* = 10.620 (5) Å	θ = 5.4–56.3°
*c* = 21.692 (12) Å	µ = 0.17 mm^−^^1^
β = 90.682 (10)°	*T* = 293 K
*V* = 2232 (2) Å^3^	Prismatic, green
*Z* = 4	0.32 × 0.27 × 0.16 mm

### Data collection

**Table:**

Bruker SMART CCD area-detector diffractometer	4257 independent reflections
Radiation source: fine-focus sealed tube	3096 reflections with *I* > 2σ(*I*)
graphite	*R*_int_ = 0.026
phi and ω scans	θ_max_ = 26.0°, θ_min_ = 1.9°
Absorption correction: multi-scan (*SADABS*; Bruker, 2002)	*h* = −11→11
*T*_min_ = 0.675, *T*_max_ = 1.000	*k* = −13→11
8761 measured reflections	*l* = −15→26

### Refinement

**Table:**

Refinement on *F*^2^	Primary atom site location: structure-invariant direct methods
Least-squares matrix: full	Secondary atom site location: difference Fourier map
*R*[*F*^2^ > 2σ(*F*^2^)] = 0.050	Hydrogen site location: inferred from neighbouring sites
*wR*(*F*^2^) = 0.155	H-atom parameters constrained
*S* = 1.01	*w* = 1/[σ^2^(*F*_o_^2^) + (0.0949*P*)^2^] where *P* = (*F*_o_^2^ + 2*F*_c_^2^)/3
4257 reflections	(Δ/σ)_max_ = 0.003
284 parameters	Δρ_max_ = 0.31 e Å^−^^3^
0 restraints	Δρ_min_ = −0.26 e Å^−^^3^

### Special details

**Table:**

Geometry. All e.s.d.'s (except the e.s.d. in the dihedral angle between two l.s. planes) are estimated using the full covariance matrix. The cell e.s.d.'s are taken into account individually in the estimation of e.s.d.'s in distances, angles and torsion angles; correlations between e.s.d.'s in cell parameters are only used when they are defined by crystal symmetry. An approximate (isotropic) treatment of cell e.s.d.'s is used for estimating e.s.d.'s involving l.s. planes.
Refinement. Refinement of *F*^2^ against ALL reflections. The weighted *R*-factor *wR* and goodness of fit *S* are based on *F*^2^, conventional *R*-factors *R* are based on *F*, with *F* set to zero for negative *F*^2^. The threshold expression of *F*^2^ > σ(*F*^2^) is used only for calculating *R*-factors(gt) *etc*. and is not relevant to the choice of reflections for refinement. *R*-factors based on *F*^2^ are statistically about twice as large as those based on *F*, and *R*- factors based on ALL data will be even larger.

### Fractional atomic coordinates and isotropic or equivalent isotropic displacement parameters (Å^2^)

**Table:**

	*x*	*y*	*z*	*U*_iso_*/*U*_eq_	
S1	0.39630 (6)	0.68577 (5)	1.02581 (2)	0.0468 (2)	
N1	0.51571 (17)	0.71961 (14)	0.92082 (7)	0.0387 (4)	
N2	0.58867 (19)	0.85690 (16)	1.00033 (8)	0.0473 (4)	
O1	0.43929 (16)	0.55550 (13)	0.86185 (6)	0.0527 (4)	
O2	0.8683 (2)	1.0158 (2)	0.86481 (10)	0.0999 (7)	
O3	0.78414 (18)	0.86963 (17)	0.80152 (7)	0.0651 (5)	
C1	0.4398 (2)	0.61190 (18)	0.91047 (9)	0.0397 (5)	
C2	0.3609 (2)	0.57762 (18)	0.96664 (9)	0.0402 (5)	
C3	0.5139 (2)	0.76745 (18)	0.97952 (9)	0.0412 (5)	
C4	0.6868 (2)	0.90740 (19)	0.95937 (10)	0.0472 (5)	
C5	0.6901 (2)	0.87651 (19)	0.89920 (10)	0.0461 (5)	
C6	0.5836 (2)	0.78885 (18)	0.87051 (9)	0.0422 (5)	
H6	0.6312	0.7281	0.8442	0.051*	
C7	0.4768 (2)	0.8581 (2)	0.83166 (9)	0.0440 (5)	
C8	0.4347 (3)	0.8122 (2)	0.77469 (10)	0.0634 (7)	
H8	0.4738	0.7389	0.7592	0.076*	
C9	0.3338 (3)	0.8758 (3)	0.74073 (11)	0.0804 (9)	
H9	0.3056	0.8445	0.7026	0.097*	
C10	0.2761 (3)	0.9832 (3)	0.76279 (13)	0.0779 (8)	
H10	0.2089	1.0253	0.7398	0.093*	
C11	0.3176 (3)	1.0286 (3)	0.81878 (13)	0.0758 (8)	
H11	0.2780	1.1018	0.8341	0.091*	
C12	0.4174 (3)	0.9670 (2)	0.85291 (11)	0.0588 (6)	
H12	0.4451	0.9996	0.8909	0.071*	
C13	0.7847 (3)	0.9961 (2)	0.99124 (12)	0.0617 (6)	
H13A	0.8750	0.9871	0.9741	0.093*	
H13B	0.7883	0.9770	1.0345	0.093*	
H13C	0.7533	1.0811	0.9855	0.093*	
C14	0.7908 (2)	0.9298 (2)	0.85547 (12)	0.0584 (6)	
C15	0.8700 (3)	0.9143 (3)	0.75156 (13)	0.0813 (9)	
H15A	0.9639	0.9276	0.7661	0.098*	
H15B	0.8346	0.9933	0.7354	0.098*	
C16	0.8664 (4)	0.8162 (4)	0.70314 (14)	0.1146 (14)	
H16A	0.9010	0.7385	0.7198	0.172*	
H16B	0.9227	0.8420	0.6693	0.172*	
H16C	0.7730	0.8045	0.6889	0.172*	
C17	0.2755 (2)	0.47861 (18)	0.96532 (9)	0.0441 (5)	
H17	0.2744	0.4352	0.9281	0.053*	
C18	0.1845 (2)	0.42670 (18)	1.01130 (9)	0.0431 (5)	
C19	0.0866 (2)	0.33502 (19)	0.99376 (10)	0.0473 (5)	
C20	−0.0026 (2)	0.2895 (2)	1.03827 (11)	0.0540 (6)	
H20	−0.0680	0.2298	1.0265	0.065*	
C21	0.0007 (2)	0.3284 (2)	1.09903 (11)	0.0536 (6)	
C22	0.0988 (3)	0.4159 (2)	1.11590 (11)	0.0581 (6)	
H22	0.1048	0.4426	1.1567	0.070*	
C23	0.1885 (2)	0.4647 (2)	1.07309 (10)	0.0531 (6)	
H23	0.2533	0.5243	1.0856	0.064*	
C24	0.0761 (3)	0.2857 (2)	0.92891 (11)	0.0661 (7)	
H24A	0.0026	0.2253	0.9261	0.099*	
H24B	0.1615	0.2461	0.9180	0.099*	
H24C	0.0575	0.3541	0.9011	0.099*	
C25	−0.1005 (3)	0.2774 (3)	1.14450 (13)	0.0756 (8)	
H25A	−0.1877	0.3191	1.1390	0.113*	
H25B	−0.0666	0.2920	1.1856	0.113*	
H25C	−0.1121	0.1886	1.1380	0.113*	

### Atomic displacement parameters (Å^2^)

**Table:**

	*U*^11^	*U*^22^	*U*^33^	*U*^12^	*U*^13^	*U*^23^
S1	0.0540 (4)	0.0431 (3)	0.0435 (3)	−0.0012 (2)	0.0112 (2)	−0.0006 (2)
N1	0.0400 (10)	0.0354 (8)	0.0408 (8)	−0.0022 (7)	0.0049 (7)	0.0019 (7)
N2	0.0497 (11)	0.0405 (9)	0.0519 (10)	−0.0045 (8)	0.0026 (8)	−0.0055 (8)
O1	0.0612 (10)	0.0505 (8)	0.0468 (8)	−0.0109 (8)	0.0111 (7)	−0.0067 (7)
O2	0.0972 (16)	0.0951 (15)	0.1080 (16)	−0.0585 (13)	0.0217 (13)	−0.0015 (12)
O3	0.0577 (11)	0.0771 (11)	0.0608 (10)	−0.0177 (9)	0.0164 (8)	0.0117 (9)
C1	0.0376 (12)	0.0378 (11)	0.0438 (10)	0.0017 (9)	0.0021 (9)	0.0017 (8)
C2	0.0400 (12)	0.0369 (10)	0.0439 (10)	0.0044 (9)	0.0060 (9)	0.0021 (8)
C3	0.0415 (12)	0.0366 (10)	0.0456 (10)	0.0046 (9)	0.0022 (9)	−0.0005 (8)
C4	0.0413 (12)	0.0371 (11)	0.0632 (13)	−0.0003 (9)	−0.0019 (10)	−0.0001 (9)
C5	0.0373 (12)	0.0406 (11)	0.0603 (13)	−0.0037 (9)	0.0015 (10)	0.0049 (9)
C6	0.0418 (12)	0.0397 (10)	0.0452 (10)	−0.0054 (9)	0.0100 (9)	0.0007 (8)
C7	0.0407 (12)	0.0499 (12)	0.0415 (10)	−0.0111 (10)	0.0068 (9)	0.0074 (9)
C8	0.0696 (18)	0.0719 (16)	0.0489 (12)	−0.0138 (14)	0.0042 (12)	−0.0001 (11)
C9	0.081 (2)	0.114 (2)	0.0459 (13)	−0.025 (2)	−0.0126 (13)	0.0155 (15)
C10	0.0650 (18)	0.099 (2)	0.0694 (17)	−0.0022 (17)	−0.0061 (14)	0.0363 (16)
C11	0.0722 (19)	0.0745 (18)	0.0805 (18)	0.0164 (15)	−0.0016 (15)	0.0167 (14)
C12	0.0576 (15)	0.0614 (15)	0.0575 (13)	0.0065 (12)	−0.0022 (11)	0.0037 (11)
C13	0.0550 (15)	0.0507 (13)	0.0792 (16)	−0.0102 (11)	−0.0052 (12)	−0.0043 (12)
C14	0.0442 (14)	0.0568 (14)	0.0743 (16)	−0.0075 (12)	0.0021 (12)	0.0128 (12)
C15	0.0662 (18)	0.104 (2)	0.0741 (17)	−0.0152 (16)	0.0208 (14)	0.0375 (16)
C16	0.136 (3)	0.149 (3)	0.0596 (17)	−0.023 (3)	0.036 (2)	0.0160 (19)
C17	0.0451 (12)	0.0391 (11)	0.0481 (11)	0.0002 (9)	0.0053 (9)	0.0026 (9)
C18	0.0418 (12)	0.0353 (10)	0.0522 (11)	0.0026 (9)	0.0071 (9)	0.0063 (9)
C19	0.0460 (13)	0.0367 (10)	0.0593 (12)	0.0020 (9)	0.0045 (10)	0.0072 (9)
C20	0.0431 (13)	0.0434 (12)	0.0756 (15)	−0.0036 (10)	0.0054 (11)	0.0103 (11)
C21	0.0441 (14)	0.0498 (13)	0.0672 (14)	0.0066 (11)	0.0131 (11)	0.0167 (11)
C22	0.0598 (15)	0.0613 (14)	0.0533 (13)	−0.0009 (12)	0.0116 (11)	0.0082 (11)
C23	0.0547 (14)	0.0499 (12)	0.0548 (12)	−0.0096 (11)	0.0084 (10)	0.0041 (10)
C24	0.0727 (18)	0.0569 (14)	0.0688 (15)	−0.0175 (13)	0.0074 (13)	−0.0034 (12)
C25	0.0597 (17)	0.0840 (18)	0.0836 (18)	−0.0012 (15)	0.0216 (14)	0.0293 (15)

### Geometric parameters (Å, °)

**Table:**

S1—C2	1.753 (2)	C12—H12	0.9300
S1—C3	1.757 (2)	C13—H13A	0.9600
N1—C3	1.371 (3)	C13—H13B	0.9600
N1—C1	1.377 (3)	C13—H13C	0.9600
N1—C6	1.477 (2)	C15—C16	1.480 (4)
N2—C3	1.274 (3)	C15—H15A	0.9700
N2—C4	1.415 (3)	C15—H15B	0.9700
O1—C1	1.213 (2)	C16—H16A	0.9600
O2—C14	1.198 (3)	C16—H16B	0.9600
O3—C14	1.334 (3)	C16—H16C	0.9600
O3—C15	1.454 (3)	C17—C18	1.448 (3)
C1—C2	1.491 (3)	C17—H17	0.9300
C2—C17	1.338 (3)	C18—C23	1.400 (3)
C4—C5	1.347 (3)	C18—C19	1.409 (3)
C4—C13	1.500 (3)	C19—C20	1.390 (3)
C5—C14	1.481 (3)	C19—C24	1.504 (3)
C5—C6	1.518 (3)	C20—C21	1.381 (3)
C6—C7	1.517 (3)	C20—H20	0.9300
C6—H6	0.9800	C21—C22	1.376 (3)
C7—C12	1.374 (3)	C21—C25	1.501 (3)
C7—C8	1.385 (3)	C22—C23	1.380 (3)
C8—C9	1.392 (4)	C22—H22	0.9300
C8—H8	0.9300	C23—H23	0.9300
C9—C10	1.360 (4)	C24—H24A	0.9600
C9—H9	0.9300	C24—H24B	0.9600
C10—C11	1.363 (4)	C24—H24C	0.9600
C10—H10	0.9300	C25—H25A	0.9600
C11—C12	1.377 (3)	C25—H25B	0.9600
C11—H11	0.9300	C25—H25C	0.9600
			
C2—S1—C3	91.59 (10)	H13B—C13—H13C	109.5
C3—N1—C1	116.53 (16)	O2—C14—O3	122.5 (2)
C3—N1—C6	120.82 (16)	O2—C14—C5	126.8 (2)
C1—N1—C6	122.33 (16)	O3—C14—C5	110.7 (2)
C3—N2—C4	116.34 (17)	O3—C15—C16	106.9 (2)
C14—O3—C15	118.4 (2)	O3—C15—H15A	110.3
O1—C1—N1	123.26 (18)	C16—C15—H15A	110.3
O1—C1—C2	126.39 (19)	O3—C15—H15B	110.3
N1—C1—C2	110.34 (16)	C16—C15—H15B	110.3
C17—C2—C1	119.81 (18)	H15A—C15—H15B	108.6
C17—C2—S1	130.25 (16)	C15—C16—H16A	109.5
C1—C2—S1	109.90 (14)	C15—C16—H16B	109.5
N2—C3—N1	126.29 (19)	H16A—C16—H16B	109.5
N2—C3—S1	122.38 (16)	C15—C16—H16C	109.5
N1—C3—S1	111.30 (15)	H16A—C16—H16C	109.5
C5—C4—N2	122.71 (19)	H16B—C16—H16C	109.5
C5—C4—C13	125.3 (2)	C2—C17—C18	131.74 (19)
N2—C4—C13	112.00 (19)	C2—C17—H17	114.1
C4—C5—C14	123.4 (2)	C18—C17—H17	114.1
C4—C5—C6	121.52 (19)	C23—C18—C19	117.94 (19)
C14—C5—C6	114.95 (19)	C23—C18—C17	122.7 (2)
N1—C6—C7	110.19 (17)	C19—C18—C17	119.39 (19)
N1—C6—C5	108.00 (16)	C20—C19—C18	118.4 (2)
C7—C6—C5	112.74 (17)	C20—C19—C24	119.5 (2)
N1—C6—H6	108.6	C18—C19—C24	122.1 (2)
C7—C6—H6	108.6	C21—C20—C19	123.4 (2)
C5—C6—H6	108.6	C21—C20—H20	118.3
C12—C7—C8	118.4 (2)	C19—C20—H20	118.3
C12—C7—C6	120.47 (18)	C22—C21—C20	117.6 (2)
C8—C7—C6	121.1 (2)	C22—C21—C25	121.6 (2)
C7—C8—C9	119.9 (3)	C20—C21—C25	120.7 (2)
C7—C8—H8	120.0	C21—C22—C23	120.9 (2)
C9—C8—H8	120.0	C21—C22—H22	119.6
C10—C9—C8	120.7 (3)	C23—C22—H22	119.6
C10—C9—H9	119.7	C22—C23—C18	121.7 (2)
C8—C9—H9	119.7	C22—C23—H23	119.1
C9—C10—C11	119.5 (3)	C18—C23—H23	119.1
C9—C10—H10	120.3	C19—C24—H24A	109.5
C11—C10—H10	120.3	C19—C24—H24B	109.5
C10—C11—C12	120.6 (3)	H24A—C24—H24B	109.5
C10—C11—H11	119.7	C19—C24—H24C	109.5
C12—C11—H11	119.7	H24A—C24—H24C	109.5
C7—C12—C11	120.9 (2)	H24B—C24—H24C	109.5
C7—C12—H12	119.5	C21—C25—H25A	109.5
C11—C12—H12	119.5	C21—C25—H25B	109.5
C4—C13—H13A	109.5	H25A—C25—H25B	109.5
C4—C13—H13B	109.5	C21—C25—H25C	109.5
H13A—C13—H13B	109.5	H25A—C25—H25C	109.5
C4—C13—H13C	109.5	H25B—C25—H25C	109.5
H13A—C13—H13C	109.5		
			
C3—N1—C1—O1	−175.24 (19)	N1—C6—C7—C8	−102.7 (2)
C6—N1—C1—O1	11.3 (3)	C5—C6—C7—C8	136.6 (2)
C3—N1—C1—C2	5.0 (2)	C12—C7—C8—C9	−0.5 (3)
C6—N1—C1—C2	−168.54 (17)	C6—C7—C8—C9	178.3 (2)
O1—C1—C2—C17	−2.7 (3)	C7—C8—C9—C10	0.2 (4)
N1—C1—C2—C17	177.07 (17)	C8—C9—C10—C11	−0.1 (4)
O1—C1—C2—S1	179.15 (18)	C9—C10—C11—C12	0.2 (4)
N1—C1—C2—S1	−1.1 (2)	C8—C7—C12—C11	0.6 (3)
C3—S1—C2—C17	−180.0 (2)	C6—C7—C12—C11	−178.1 (2)
C3—S1—C2—C1	−2.08 (15)	C10—C11—C12—C7	−0.5 (4)
C4—N2—C3—N1	−2.9 (3)	C15—O3—C14—O2	−2.9 (4)
C4—N2—C3—S1	174.98 (15)	C15—O3—C14—C5	176.3 (2)
C1—N1—C3—N2	171.51 (19)	C4—C5—C14—O2	−10.0 (4)
C6—N1—C3—N2	−14.9 (3)	C6—C5—C14—O2	166.5 (2)
C1—N1—C3—S1	−6.6 (2)	C4—C5—C14—O3	170.8 (2)
C6—N1—C3—S1	166.99 (14)	C6—C5—C14—O3	−12.6 (3)
C2—S1—C3—N2	−173.40 (18)	C14—O3—C15—C16	167.7 (3)
C2—S1—C3—N1	4.81 (15)	C1—C2—C17—C18	−177.9 (2)
C3—N2—C4—C5	9.0 (3)	S1—C2—C17—C18	−0.2 (4)
C3—N2—C4—C13	−170.39 (18)	C2—C17—C18—C23	−11.2 (4)
N2—C4—C5—C14	179.09 (19)	C2—C17—C18—C19	168.2 (2)
C13—C4—C5—C14	−1.6 (4)	C23—C18—C19—C20	1.5 (3)
N2—C4—C5—C6	2.8 (3)	C17—C18—C19—C20	−177.96 (19)
C13—C4—C5—C6	−178.0 (2)	C23—C18—C19—C24	−178.5 (2)
C3—N1—C6—C7	−100.3 (2)	C17—C18—C19—C24	2.0 (3)
C1—N1—C6—C7	73.0 (2)	C18—C19—C20—C21	−0.8 (3)
C3—N1—C6—C5	23.3 (3)	C24—C19—C20—C21	179.2 (2)
C1—N1—C6—C5	−163.48 (17)	C19—C20—C21—C22	−0.6 (3)
C4—C5—C6—N1	−17.7 (3)	C19—C20—C21—C25	178.7 (2)
C14—C5—C6—N1	165.70 (18)	C20—C21—C22—C23	1.3 (3)
C4—C5—C6—C7	104.3 (2)	C25—C21—C22—C23	−178.0 (2)
C14—C5—C6—C7	−72.3 (2)	C21—C22—C23—C18	−0.6 (4)
N1—C6—C7—C12	76.0 (2)	C19—C18—C23—C22	−0.9 (3)
C5—C6—C7—C12	−44.7 (3)	C17—C18—C23—C22	178.6 (2)
